# Estimation of subjective quality of life in schizophrenic patients using speech features

**DOI:** 10.3389/fresc.2023.1121034

**Published:** 2023-03-10

**Authors:** Yuko Shibata, John Noel Victorino, Tomoya Natsuyama, Naomichi Okamoto, Reiji Yoshimura, Tomohiro Shibata

**Affiliations:** ^1^ Department of Life Science and System Engineering, Graduate School of Life Science and Systems Engineering, Kyushu Institute of Technology, Kitakyushu, Japan; ^2^ Department of Psychiatry, University of Occupational and Environmental Health, Kitakyushu, Japan

**Keywords:** quality of life, schizophrenia, speech analysis, machine learning, model development

## Abstract

**Introduction:**

Patients with schizophrenia experience the most prolonged hospital stay in Japan. Also, the high re-hospitalization rate affects their quality of life (QoL). Despite being an effective predictor of treatment, QoL has not been widely utilized due to time constraints and lack of interest. As such, this study aimed to estimate the schizophrenic patients' subjective quality of life using speech features. Specifically, this study uses speech from patients with schizophrenia to estimate the subscale scores, which measure the subjective QoL of the patients. The objectives were to (1) estimate the subscale scores from different patients or cross-sectional measurements, and 2) estimate the subscale scores from the same patient in different periods or longitudinal measurements.

**Methods:**

A conversational agent was built to record the responses of 18 schizophrenic patients on the Japanese Schizophrenia Quality of Life Scale (JSQLS) with three subscales: “Psychosocial,” “Motivation and Energy,” and “Symptoms and Side-effects.” These three subscales were used as objective variables. On the other hand, the speech features during measurement (Chromagram, Mel spectrogram, Mel-Frequency Cepstrum Coefficient) were used as explanatory variables. For the first objective, a trained model estimated the subscale scores for the 18 subjects using the Nested Cross-validation (CV) method. For the second objective, six of the 18 subjects were measured twice. Then, another trained model estimated the subscale scores for the second time using the 18 subjects' data as training data. Ten different machine learning algorithms were used in this study, and the errors of the learned models were compared.

**Results and Discussion:**

The results showed that the mean RMSE of the cross-sectional measurement was 13.433, with k-Nearest Neighbors as the best model. Meanwhile, the mean RMSE of the longitudinal measurement was 13.301, using Random Forest as the best. RMSE of less than 10 suggests that the estimated subscale scores using speech features were close to the actual JSQLS subscale scores. Ten out of 18 subjects were estimated with an RMSE of less than 10 for cross-sectional measurement. Meanwhile, five out of six had the same observation for longitudinal measurement. Future studies using a larger number of subjects and the development of more personalized models based on longitudinal measurements are needed to apply the results to telemedicine for continuous monitoring of QoL.

## 1. Introduction

The number of psychiatric beds in Japan is much larger than in other countries, and the length of hospital stay is as long as 285 days (Italy: 13.9 days, U.K.: 42.3 days) (1). The Ministry of Health, Labour, and Welfare (MHLW) has announced a vision for reforming mental health and medical welfare in response to prolonged hospitalization. The MHLW vision fundamentally shifts its policy from inpatient care to community-based care. The vision clearly states the improvement of inpatient treatment, the improvement of patients' Quality of Life (QoL), and the development of support for early discharge from the hospital (2). Furthermore, a survey of readmission rates for 24,781 patients discharged in 2014 showed that 23% were re-admitted three months after discharge, 30% six months later, and 37% one year later (3). Prolonged hospitalization and high readmission rates are issues for psychiatric care in Japan.

QoL is an effective predictor of symptom remission and functional recovery among schizophrenic patients. As such, QoL is an essential measure of outcome in treatment (4, 5). It is crucial to understand and assess fluctuations in QoL scores and routine tests such as blood sampling; then use this information in interventions (6). However, QoL assessment is not routinely performed in clinical practice due to time constraints and lack of training and interest (7–9).

QoL can be divided into objective assessment and subjective assessment. This study focuses on subjective QoL because patients are the main actors in their lives during hospitalization and after discharge. The subjective assessment is possible because schizophrenic patients can feel and report social impairment (10). In addition, this study examined the use of voice input to estimate QoL status instead of the conventional self-administered and semi-constructed interview methods. Multi-lingual speech recognition and emotional speech recognition have been actively studied in recent years (11–13). Many voice-based applications have also been developed to remotely monitor the status and characteristics of speakers, such as health status (14–16). These latest developments motivate this study to consider speech recognition as a fast and efficient means of human-machine interaction (17).

Therefore, this study examined the estimation of subscale scores that measure the subjective QoL of schizophrenic patients using speech features as a simple method to measure QoL. The objectives were to (1) estimate the subscale scores from different patients or cross-sectional measurements, and (2) estimate the subscale scores from the same patient in different periods or longitudinal measurements. The proposed method allows schizophrenic patients to measure their subjective QoL by themselves in the future. Furthermore, the proposed method provides opportunities to monitor QoL continuously and regularly during hospitalization and after discharge. Patients and medical care providers can share data and analysis.

## 2. Material and methods

We examined the feasibility of estimating the subjective QoL of schizophrenic patients using speech features. The three subscale scores of the Japanese Schizophrenia Quality of Life Scale (JSQLS) were collected using a conversational agent. The conversational agent recorded the JSQLS responses and the audio of the conversation. Then, models were developed and compared to estimate the subscale scores. There were two kinds of models developed in this study.

1) Model development to estimate subscale scores from among different patients or cross-sectional measurements
2) Model development to estimate subscale scores from the same patient in different periods or longitudinal measurements

### 2.1. Target population demographics

Eighteen schizophrenic patients who agreed to participate in the study were included (Table 1). The mean age was 47.17 years, with seven males and 11 females. Global Assessment of Functioning (GAF) is a scale used to assess an overview of a subject's functioning. Psychological, social, and occupational functioning is rated as a single variable on an integer scale of 1–100 points (18). The rater evaluates the subject's condition according to the scale's rating criteria. For example, a 91–100 score indicates “very good functioning and no psychiatric symptoms.” Higher scores mean better symptoms and functioning. In this study, the psychiatrist or nurse in charge of the patient performed the evaluation (Table 2).

**Table 1:** Subjects’ demographics

Characteristics	Subjects
Sample size, *n*	18.000
Age, mean (Std. Dev.)	45.170 (16.576)
Male sex, *n* (%)	7.000 (38.890)
GAF, Range	32.000–70.000

**Table 2:** Demographics of subjects measured twice.

Characteristics	Subjects
Sample size, *n*	6.000
Age, mean (Std. Dev.)	32.160 (6.150)
Male sex, *n* (%)	1.000 (16.670)
GAF, Range	50.000–70.000

### 2.2. SQLS as a measure of subjective QoL

JSQLS was used to measure subjective QoL. The JSQLS provides a subjective assessment of the impact of the disease on the subject's life. The JSQLS consists of three scales: Psychosocial (15 items), Motivation and Energy (7 items), and Symptoms and Side-effects (8 items). During the scale development in previous studies, the questions were selected based on in-depth patient interviews. Then, the JSQLS questions were examined for reliability and validity (18, 19).

#### 2.2.1. JSQLS calculation method

This section describes how the three subscale scores are calculated and evaluated based on the subject's answers. There are five options for each question, and the score for each question ranges from 0 to 4 points.

“Always” (4 points)
“Often” (3 points)
“Sometimes” (2 points)
“Rarely” (1 point)
“Never” (0 points)

Each subscale was calculated to take values between 0 and 100, with higher scores indicating worse QoL while lower scores indicating better QoL.

(1) $$\begin{array}{rl} & \mathrm{Score}\;\mathrm{of}\;\mathrm{each}\;\mathrm{subscale}\;\\ & =\;\frac{\mathrm{Sum}\;\;\mathrm{of}\;\mathrm{the}\;\mathrm{crude}\;\mathrm{scores}\;\mathrm{for}\;\mathrm{each}\;\mathrm{scale}}{4\;\times \;\mathrm{Number}\;\mathrm{of}\;\mathrm{questions}\;\mathrm{for}\;\mathrm{each}\;\mathrm{scale}}\;\times \;100 \end{array}$$

On the one hand, the numerator is the total score based on each subject's choices. The denominator is calculated as 4 × 15 questions for “Psychosocial,” 4 × 7 questions for “Motivation and Energy,” and 4 × 8 questions for “Symptoms and Side-effects.” Note that four questions under “Motivation and Energy” are scored inversely, i.e., “Always” (0 points), “Often” (1 point), “Sometimes” (2 points), “Rarely” (3 points), and “never” (4 points).

### 2.3. Development of a conversational agent to measure subjective QoL

**Figure 1:**
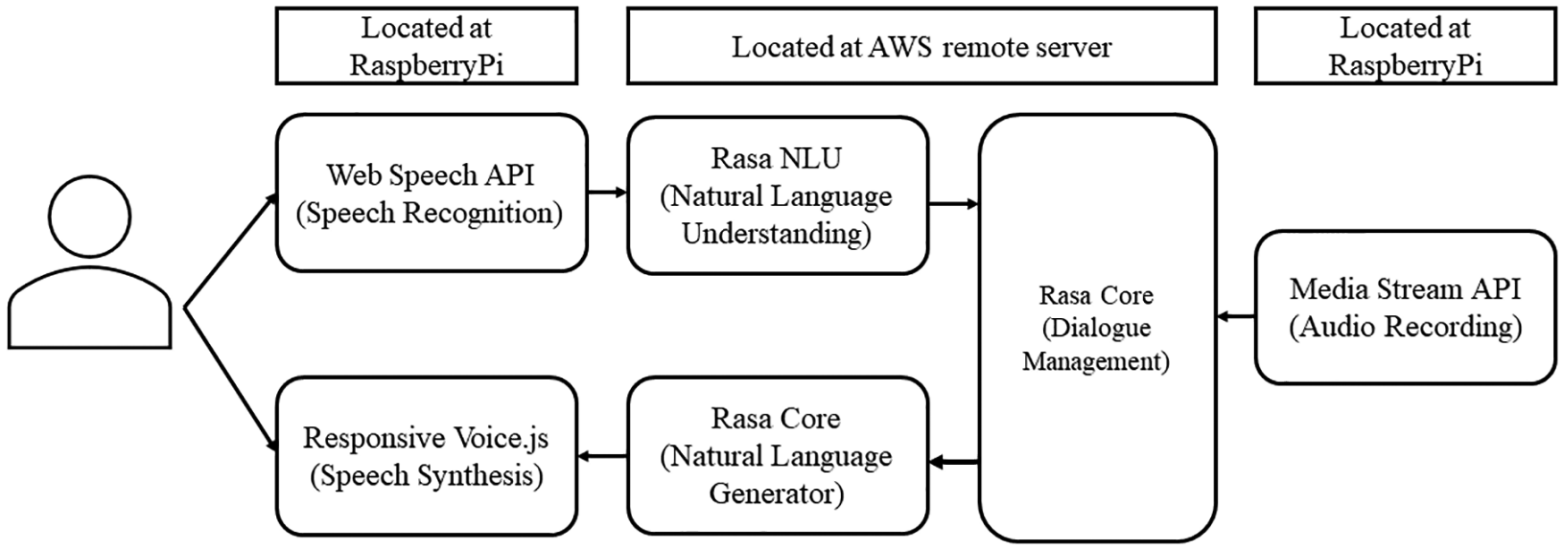
Conversational agent system architecture.

In this study, the conversational agent asked the patient 30 JSQLS questions and recorded the subject's voice as he or she answered each question (Figure 1).

The Web Speech API converts the participant's speech into text. Then, the conversation agent uses natural language understanding to classify the answer choices (“Always,” “Often,” “Sometimes,” “Rarely,” and “Never”). Next, Rasa Core (20) manages the interaction, including the flow of conversation and context processing. Rasa Core's natural language generator selects appropriate text responses based on the context and flow of the conversation. Finally, ResponsiveVoice.js generates the spoken response from the text response.

### 2.4. Measurement method

**Figure 2:**
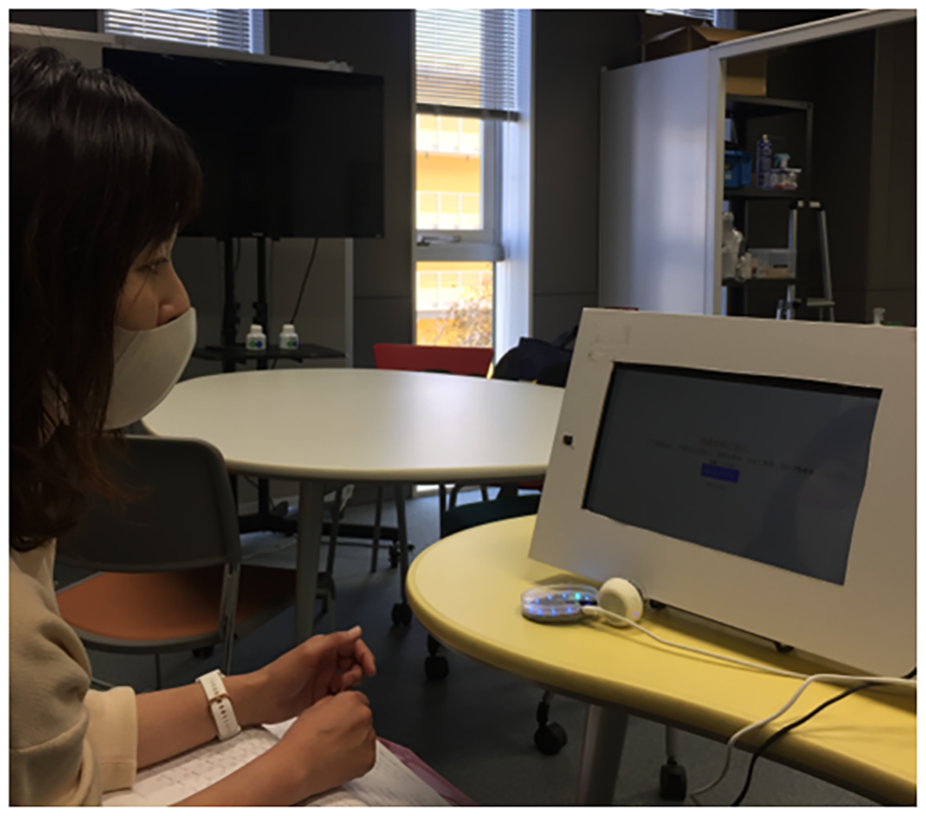
Preliminary experiment setup.

Measurements were taken at the subject's hospital, a continuous employment support facility, and the subject's home. A quiet environment was ensured during the measurement for voice interaction and recording. First, the subjects were asked to read the 30 JSQLS questions before the measurement with the conversation agent. This step was implemented to prepare the subjects with the subsequent questions and clarify any questions. Then, the conversation agent spoke and displayed a question for the subject to listen and to see, respectively (Figure 2). Next, the subject answered back to the conversation agent. The conversation agent recorded the subject's response and audio using a microphone array. From this point, the conversation agent either (A) repeats the question if the subject's response is not understood or (B) proceeds to the next question until all 30 questions are finished (Figure 2).

### 2.5. Audio processing and speech feature extraction

The following describes the acquired speech data. Subjects' speech was recorded at a sampling frequency of 48 kHz; the total unedited recording time, including conversational agent announcements, for the 18 subjects’ speech data was 98.200 min, with an average recording time of 5.456 min. The shortest recording time was 3.600 min and the longest was 10.917 min. The speech for the analysis was stripped of the conversational agent's announcements, silences, false responses, and noises. When the subject's speech was unclear, the conversation agent would listen back to the subject's speech, resulting in individual differences in recording time. The total recording time after removing the conversational agent's voice and the noise was 18.200 min, with an average duration of 1.011 min.

**Figure 3:**
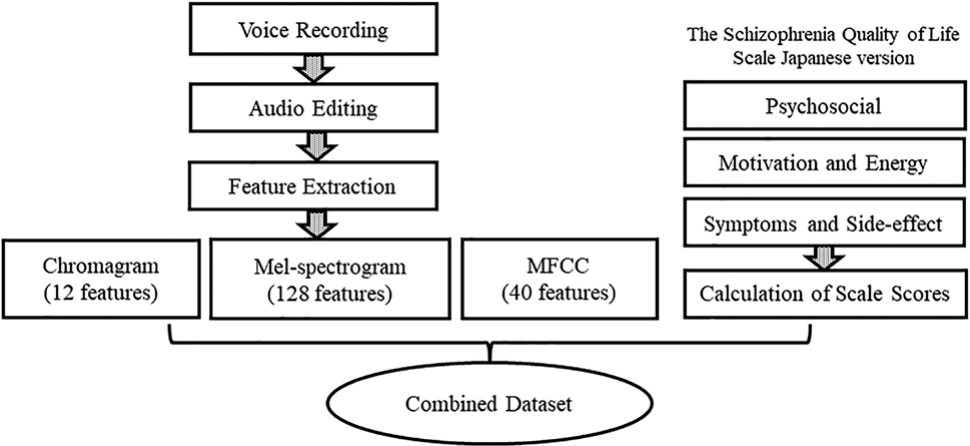
Data processing.

Using the Librosa audio library, speech data from 18 subjects were input, and Mel-spectrogram (128 dimensions), Mel-Frequency Cepstrum Coefficient (MFCC) (40 dimensions), and Chromagram (12 dimensions) speech features were Chromagram (12 dimensions) were extracted (Figure 3). A total of 3,240 speech features with 180 dimensions per subject and 18 subjects were used as the objective variables. Mel spectrogram and MFCC mimicked, to an extent, the natural sound frequency reception pattern of humans (13) and are often used for voice separation and classification (21). Chromagram can infer the vocal tract's resonance characteristics as the signal's energy distribution concerning saturation and time (22).

### 2.6. Model development

A model was developed using the extracted speech features to estimate the three JSQLS subscale scores. The features used to develop the model are the following.

Let *x* be the explanatory variable and *y*. be the objective variable. Specifically,

$x_{1∼12}$: Chromagram
$x_{1∼128}$: Mel-spectrogram
$x_{1∼40}$: MFCC
$y_{1}$: “Psychosocial” subscale
$y_{2}$: “Motivation and Energy” subscale
$y_{3}$: “Symptoms and Side-effects” subscale

Python libraries like Pandas and Sklearn were used for data processing and model development. In this study, ten machine learning algorithms were utilized, and the errors of the trained models were compared. Ridge Regression, Lasso Regression, Elastic-Net Regression, k-Nearest Neighbors (k-NN), Decision Tree (DT), Support Vector Regression (SVR), Linear SVR (L.SVR), Random Forest (RF), Gradient Boosting (GB), and AdaBoost algorithms, were considered for developina model in estimating the subjective QoL of the subjects. Each algorithm is described below.

#### 2.6.1. Ridge regression

Ridge Regression is a parameter estimation method used to address that addresses the collinearity problem frequently arising in multiple linear regression (23). Ridge Regression's coefficients minimize the sum of squared penalized residuals (24). L2 regularization is used in Ridge Regression.

$$\underset{\omega }{\min}||X\omega -y|{|}_{2}^{2}+\alpha ||\omega |{|}_{2}^{2}$$

#### 2.6.2. Lasso regression

Lasso Regression minimizes the residual sum of squares subject to the sum of the absolute value of the coefficients being less than a constant. Because of the nature of this constraint, it tends to produce some coefficients that are exactly 0 and hence gives interpretable models (25). L1 regularization is used in Lasso Regression. The objective function to minimize is (26):

(2) $$\underset{w}{\min}\frac{1}{2n_{\mathrm{samples}}}||Xw-y|{|}_{2}^{2}+\alpha ||w|{|}_{1}$$

#### 2.6.3. Elastic-Net regression

The Elastic-Net is particularly useful when the number of predictors (*p*) is much bigger than the number of observations (*n*). In contrast, the Lasso Regression does not have a satisfactory variable selection method to handle the *p >* *n* case. Therefore, Elastic-Net was proposed as an improved version of Lasso Regression to analyze high-dimensional data. The L1 part of the Elastic-Net performs automatic variable selection, while the L2 part stabilizes the solution paths. Hence, this method improves the prediction (27). The objective function to be minimized is (28):

(3) $$\underset{w}{\min}\frac{1}{2n_{\mathrm{samples}}}||Xw-y|{|}_{2}^{2}+\alpha \rho ||w|{|}_{1}+\frac{\alpha (1-\rho )}{2}||w|{|}_{2}^{2}$$

#### 2.6.4. k-Nearest neighbors (k-NN)

k-NN algorithm for regression is a supervised learning approach. It predicts the target based on the similarity with other available cases. The similarity is calculated using the distance measure, with Euclidian distance being the most common approach. Predictions are made by finding the *k* most similar instances, i.e., the neighbors, of the testing point, from the entire dataset (29).

#### 2.6.5. Decision tree (DT)

The Decision Trees algorithm is a non-parametric supervised learning method used for classification and regression.

In Decision Trees, a hierarchical tree structure consisting of Yes-No questions is learned. The disadvantage of decision trees is that they are prone to over-fitting and tend to be less versatile (30).

#### 2.6.6. Support vector regression (SVR)

Instead of minimizing the observed training error, Support Vector Regression (SVR) attempts to minimize the generalization error bound to achieve generalized performance. SVR's concept is based on the computation of a linear regression function in a high-dimensional feature space where the input data are mapped *via* a nonlinear function (31).

#### 2.6.7. Linear SVR (L.SVR)

Support Vector Regression (SVR) and Support Vector Classification (SVC) are time-consuming when using kernels. It has been demonstrated that Linear SVC and L. SVR generate models equivalent to kernel-SVR efficiently (32).

#### 2.6.8. Random forest (RF)

Random Forest is one of the methods to deal with the problem of over-fitting to the training data in the DT algorithm. RFs consist of tree-structured classifiers {*h* (x, k), k = 1, …} where the {k} are identically independent distributed random vectors. Each tree cats a vote for the most popular class at input *x* (33).

#### 2.6.9. Gradient boosting (GB)

The Gradient Boosting algorithm constructs additive regression models by sequentially fitting a simple parameterized function (base learner) to current “pseudo”-residuals by least squares at each iteration. The execution speed and approximation accuracy of GB can be greatly improved by incorporating randomization into the procedure (34).

#### 2.6.10. Adaboost

Boosting is an approach to machine learning based on combining many relatively weak and i inaccurate rules to create a highly accurate prediction rule (35). The core principle of the AdaBoost regressor is to learn a sequence of weak regressors with high bias error but with low variance error. Repeatedly reweighted training instances are done based on the prediction error of each boosting iteration (36).

#### 2.6.11. Nested cross-validation approach

**Figure 4:**
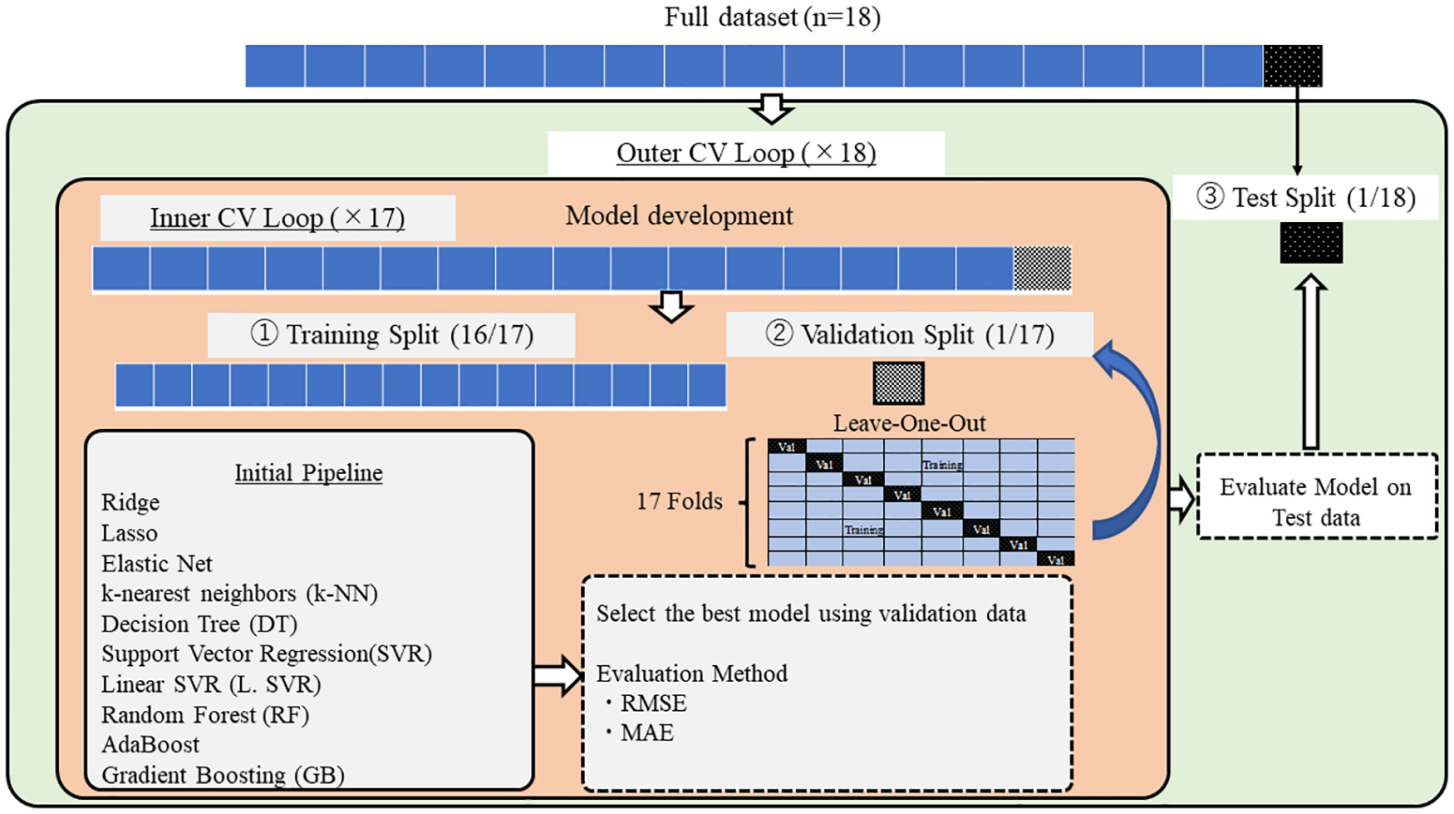
Model development.

The Nested Cross-validation (CV) approach was used to compare machine learning algorithms on smaller subsets of the dataset (37) (Figure 4). Conventional CV uses the same data to compare different algorithms and evaluate the model's performance. The conventional CV approach leads to data leakage and over-fitting. On the other hand, the nested CV splits the data into training (①), validation (②), and test sets (③) multiple times. First, the outer loop divides the entire dataset into train and test sets. The outer loop is in charge of evaluating the model performance using the test set (③). Then, the inner loop divides the train set further into smaller training (①) and validation sets (②). In the inner loop, the best model was selected among the ten algorithms by comparing the average RMSE and MAE values. The Leave-One-Out method was used for partitioning the dataset. The default hyperparameters for each algorithm were kept during the entire model development. These default hyperparameters were provided in the Scikit learn library (see Appendix).

For each of the ten algorithms, the error between ground truth and validation data was calculated using RMSE (Root Mean Squared Error) and MAE (Mean Absolute Error). RMSE is characterized by a strict evaluation of the error between the ground truth and the estimate using the squared form. The lower the RMSE is, the better the estimates of the model. On the other hand, MAE is the mean of the absolute difference between the ground truth and the estimated values. The lower the MAE is, the better the estimates of the model.

RMSE is computed as follows.

(4) $$RMSE\;=\;\sqrt{\frac{1}{n}\sum_{i=0}^{n-1}{\left(y_{i}-\hat{y_{i}}\right)}^{2}}$$

On the other hand, MAE is calculated as follows.

(5) $$MAE\;=\;\frac{1}{n}\sum_{i=1}^{n}|y_{i}-\hat{y_{i}}|$$

where *n* is the total number of data, $y_{i}$ is the actual value, ${\hat{y}}_{i}$ denotes the predicted value. Since this study estimates the subjective QoL thru three subscale scores, the average RMSE and MAE over these three subscale scores were also calculated. The average RMSE and MAE over three subscales were used during the model comparison.

### 2.7. Speech feature importance by SHAP value

In recent years, the interpretability of models has become more important than their accuracy. SHApley Additive exPlanations (SHAP) is a unified framework for interpreting predictions, allowing us to understand each feature's importance for the prediction (38).

Therefore, in this study, the SHAP value helps identify which of the three speech features contributes to the model.

### 2.8. Model development and evaluation to estimate scale scores from longitudinal measurements

QoL scores are inferred to change over time depending on the subject's condition. Therefore, we selected six subjects out of 18 subjects and conducted the second measurement after an average of 54.333 days (S.D. = 24.426). The data from the first 18 subjects were used as training data. For each of the ten models (using the same machine learning algorithm as in 2.6), the mean RMSE values for the three scales were compared using the validation data. The best model was used. Next, we evaluated the model using the scale scores of the six participants as unseen test data.

## 3. Results

This section describes the best models and evaluations of the ten algorithms selected for the cross-sectional and longitudinal measurements. Finally, we discuss the speech features that contributed to the model development.

### 3.1. Model comparison on validation set for cross-sectional measurement

First, the mean subscale scores among the 18 subjects (Table 3) were 45.000 for the “Psychosocial” subscale, 49.389 for the “Motivation and Energy” subscale, and 27.944 for the “Symptoms and Side-effects” subscale. These subscale scores were obtained from the subjects' JSQLS responses. The last subscale had the lowest score among the three subscales, which suggests that the subjects of this study had a good QoL concerning their symptoms and subsequent side effects. However, the minimum and maximum scores for the “Symptoms and Side Effects” scale were 0 and 78, respectively, indicating significant individual differences.

**Table 3:** RMSE and MAE scores for each scale on cross-sectional measurements.

Scale	Mean	Std. Dev.	Variance	Median	Range
Psychosocial	45.000	17.057	274.778	46.000	15.000–80.000
Motivation and Energy	49.389	16.288	250.571	50.000	21.000–82.000
Symptoms and Side-effects	27.944	18.031	307.053	23.500	0.000–78.000

Then, the ten algorithms were compared using the validation set produced in the inner loop. The mean RMSE and MAE values were calculated and ranked. With this method, the trained k-NN algorithm produced the lowest mean RMSE and MAE of 13.433 (SD = 10.206, *n* = 18). The average RMSE and MAE values for the validation data of the other models were in the following order (the mean values of RMSE and MAE are equal, and therefore one value is shown): SVR: 13.697, RF: 13.697, GB: 14.263, AdaBoost: 14.663, DT: 15.973, L.SVR: 21.466, Ridge: 21.496, ElasticNet: 25.976, Lasso: 28.975 (Table 4).

**Table 4:** The average RMSE and MAE values for the validation data.

Models	Average RMSE and MAE scores	S.D.
K-NN	13.443	10.206
SVR	13.697	9.580
RF	13.697	9.346
GB	14.263	7.715
AdaBoost	14.663	9.391
DT	15.973	8.156
L. SVR	21.466	12.217
Ridge	21.496	12.242
Elastic-Net	25.976	14.460
Lasso	28.975	18.129

### 3.2. Model comparison on test set for cross-sectional measurement

The selected best model (k-NN) was evaluated using the test set produced in the outer loop. The training resulted in a mean RMSE of 14.361 (SD = 0.674, *n* = 18) and a mean MAE of 10.9510 (SD = 0.6347, *n* = 18). The trained k-NN model produced a mean RMSE and MAE of 13.304 (SD = 10.392, *n* = 18) on the test set. The mean test RMSE was better than during training, and this observation can also be seen in 12 out of the 18 folds (Table 5).

**Table 5:** Evaluation of training and test data with k-NN.

Fold	1	2	3	4	5	6	7	8	9	10
Training	14.755	14.148	14.671	14.218	14.836	12.697	13.886	14.196	12.648	14.648
Test	3.267	21.133	5.933	11.800	8.467	38.200	28.333	17.000	32.867	7.133
Fold	11	12	13	14	15	16	17	18	Mean	Std. Dev.
Training	14.628	14.906	14.857	14.641	14.660	14.623	14.776	14.711	14.361	0.674
Test	14.867	3.400	6.200	12.467	9.067	7.600	6.600	5.133	13.304	10.392

*Values are shown for RMSE and MAE.

The RMSE and MAE for each subscale were 14.644 for “Psychosocial”, 13.633 for “Motivation and Energy”, and 11.633 for “Symptoms and Side-effects” (Table 6). The RMSE and MAE for “Symptoms and Side-effects” had the lowest values, but the minimum and maximum values were 1.200 and 51.800, respectively, which were larger than the other scales.

**Table 6:** RMSE and MAE scores for each subscale.

Scale	Mean	Std. Dev.	Variance	Median	Range
Psychosocial	14.644	10.568	111.691	11.100	0.200−36.600
Motivation and Energy	13.633	10.323	106.570	12.300	1.200−34.200
Symptoms and Side-effects	11.633	13.380	179.041	5.600	1.200−51.800

**Figure 5:**
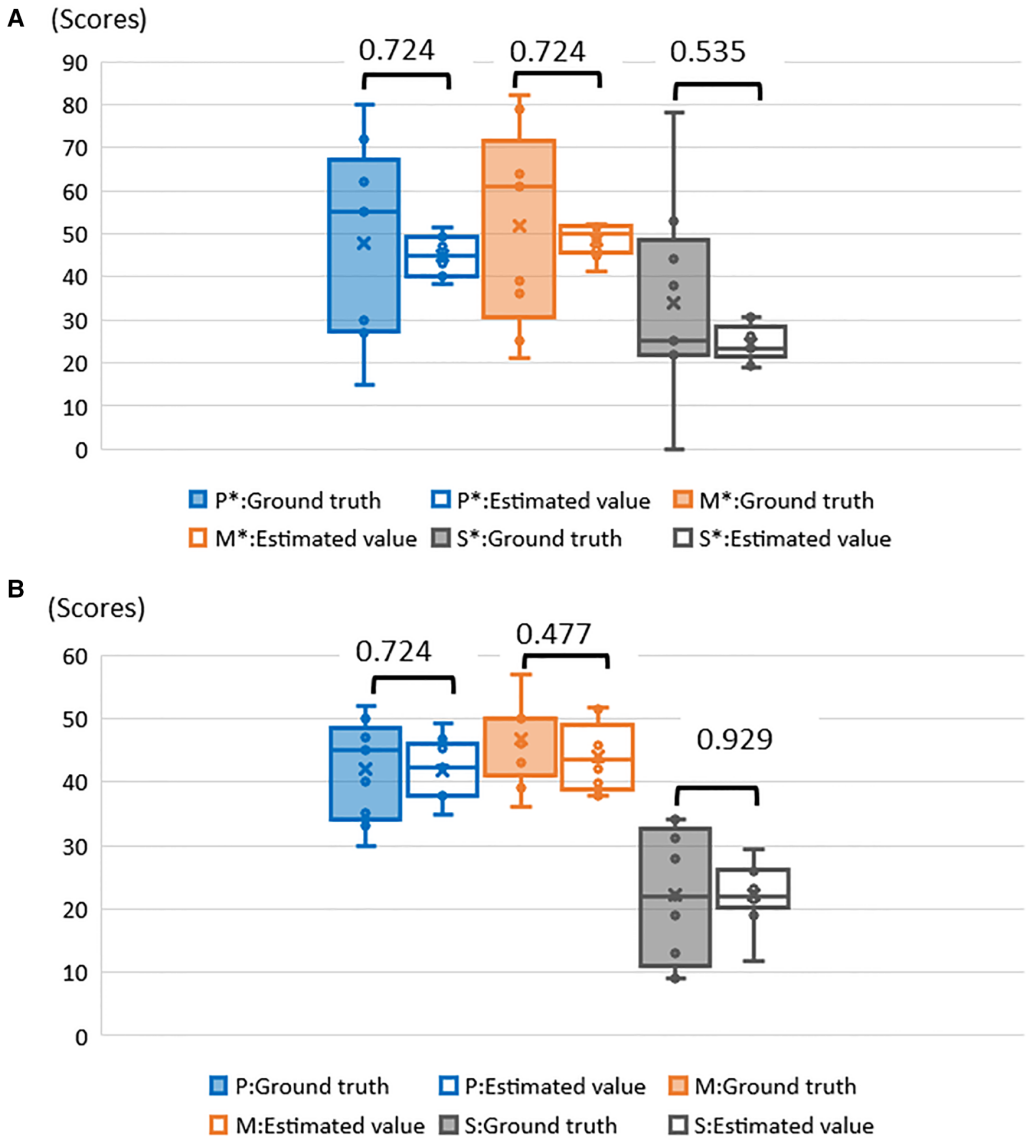
(**A**) Examination of significant differences between ground truth and estimates of RMSE and MAE above 10. *P, psychosocial; M, motivation and energy; S, symptoms and side–effects. (**B**) Examination of significant differences between ground truth and estimates of RMSE and MAE less than 10. *P, psychosocial; M, motivation and Energy; S, symptoms and side–effects.

In each fold, RMSE and MAE were above 10 in 8 folds. Among them, fold 6 had the highest RMSE and MAE (38.200) and the highest scale scores (Psychosocial: 80, Motivation and Energy: 82, Symptoms and Side-effects: 78). Similarly, fold 9 had RMSE and MAE of 32.867 and lowest scale scores (Psychosocial: 15, Motivation and Energy: 21, Symptoms and side- effects: 0) (Table 7). There were 10 folds that could be estimated with RMSE and MAE less than 10. Among them, fold 1 had the lowest value of 3.267 (Table 8). The RMSE and MAE for each fold were then divided into two groups, above and less than 10, to determine whether there was a significant difference between the ground truth and the estimates for each group; for the group with RMSE and MAE above 10 (Figure 5A), “Psychosocial” *p* = 0.724, “Motivation and Energy” *p* = 0.724, and “Symptoms and Side-effects” *p* = 0.535. In the group with RMSE and MAE less than ten (Figure 5B), “Psychosocial” *p* = 0.724, “Motivation and Energy” *p* = 0.477, “Symptoms and Side-effects” *p* = 0.929. There were no statistically significant differences between the ground truth and the estimates.

**Table 7:** Ground truth and estimated values for each subject with RMSE above 10.

Fold	Ground truth			Estimated value			RMSE	MAE
	Psychosocial	Motivation and Energy	Symptoms and Side-effects	Psychosocial	Motivation and Energy	Symptoms and Side-effects		
2	27.000	25.000	38.000	49.200	51.400	23.200	21.133	21.133
4	28.000	39.000	25.000	49.200	51.400	23.200	11.800	11.800
6	80.000	82.000	78.000	47.200	52.000	26.200	38.200	38.200
7	62.000	79.000	53.000	44.800	44.800	19.400	28.333	28.333
8	72.000	64.000	22.000	40.400	46.400	23.800	17.000	17.000
9	15.000	21.000	0.000	51.600	52.200	30.800	32.867	32.867
11	62.000	61.000	44.000	43.200	48.600	30.600	14.867	14.867
14	55.000	61.000	22.000	38.400	41.400	23.200	12.467	12.467
Median of all folds^a^	46.000	50.000	24.000					

^a^Median of all folds in all scales.

**Table 8:** Ground truth and estimated values for each subject with RMSE less than 10.

Fold	Ground truth			Estimated value			RMSE	MAE
	Psychosocial	Motivation and Energy	Symptoms and Side-effects	Psychosocial	Motivation and Energy	Symptoms and Side-effects		
1	47.000	50.000	22.000	45.200	46.400	26.400	3.267	3.267
3	50.000	43.000	19.000	37.800	39.800	21.400	5.933	5.933
5	40.000	46.000	34.000	49.200	51.400	23.200	8.467	8.467
10	30.000	50.000	13.000	38.000	37.800	11.800	7.133	7.133
12	47.000	57.000	34.000	46.800	51.600	29.400	3.400	3.400
13	52.000	50.000	31.000	42.800	45.800	25.800	6.200	6.200
15	30.000	36.000	22.000	40.000	50.000	18.800	9.067	9.067
16	45.000	36.000	9.000	38.200	42.000	19.000	7.600	7.600
17	35.000	50.000	28.000	42.400	43.600	22.000	6.600	6.600
18	33.000	39.000	9.000	34.800	37.800	21.400	5.133	5.133
Median of all folds^a^	46.000	50.000	24.000					

^a^Median of all scale scores.

### 3.3. Model comparison on validation set for longitudinal (model to estimate scores for six longitudinal measurements)

First, the mean subscale scores among the six subjects were 46.500 for the “Psychosocial” subscale, 47.667 for the “Motivation and Energy” subscale, and 25.000 for the “Symptoms and Side-effects” subscale. Similar to the results of the first measurement, the scores on the “Symptoms and Side-effects” subscale were the lowest.

The models were developed using data from 18 subjects in order to estimate the scale scores for the 6 subjects. Ten algorithms were then compared using the validation set created in the inner loop. Mean RMSE and MAE values were computed and ranked. Thus, the trained RF algorithm produced the lowest mean RMSE and MAE of 13.301 (SD = 8.870, *n* = 18). The mean RMSE and MAE for the validation data of the other models were in the following order (mean RMSE and MAE are equal and represent a single value): k-NN: 13.304, SVR: 13.537, GB: 13.832, AdaBoost: 15.090, DT: 15.648, L. SVR: 20. 630, Ridge: 20.637, Elastic-Net: 26.105, Lasso: 30.787.

### 3.4. Model comparison on test set for longitudinal measurement

The RMSE and MAE for each subscale were 9.607 for the “Psychosocial” subscale, 4.767 for the “Motivation and Energy” subscale, and 9.508 for the “Symptoms and Side-effects” subscale (Table 9). The minimum and maximum values of RMSE and MAE for the “Psychosocial” subscale were 2.030 and 17.000, respectively, which were larger than the other scales.

**Table 9:** RMSE and MAE scores for each scale on longitudinal measurements.

Scale	Mean	Std. Dev.	Variance	Median	Range
Psychosocial	9.607	6.226	38.757	10.085	2.030–17.000
Motivation and Energy	4.767	3.990	15.924	5.020	0.140–9.060
Symptoms and Side-effects	9.508	5.734	32.883	8.465	2.770–17.050

The results of the first and second measurements for the six subjects showed that the scores for each scale varied between −18 and +14 from the first measurement (Table 10). The RMSE and MAE values for each fold, five out of 6 folds, were less than 10. Fold 3 had the lowest RMSE and MAE at 4.557 and fold 2 had the highest at 13.540. The three subscales of Fold 2 remained high in the two measurements.

**Table 10:** First and second measurements (ground truth), and estimation scores^a^ from longitudinal measurements.

Fold	First measurement			Second measurement			RMSE	MAE
	Psychosocial	Motivation and Energy	Symptoms and Side-effects	Psychosocial	Motivation and Energy	Symptoms and Side-effects		
1	30.000	50.000	13.000	37.000 (+7)	36.000 (+14)	22.000 (+9)	7.380	7.380
2	62.000	61.000	44.000	60.000 (−2)	54.000 (−7)	47.000 (+3)	13.540	13.540
3	47.000	57.000	34.000	40.000 (−7)	54.000 (−3)	25.000 (−9)	4.577	4.577
4	52.000	50.000	31.000	35.000 (−17)	46.000 (−4)	28.000 (−3)	6.513	6.513
5	55.000	61.000	22.000	58.000 (+3)	43.000 (−18)	16.000 (−6)	8.360	8.360
6	45.000	36.000	9.000	47.000 (+2)	43.000 (+7)	3.000 (−6)	7.393	7.393
Median	50.000	54.000	27.000	44.000	45.000	24.000		

^a^RMSE and MAE are calculated from the ground truth and estimated values of the second measurement.

### 3.5. Speech feature importance to the estimation of scale scores

Speech features contributing to scoring estimation for each scale were identified by SHAP values. MFCC1 was selected as the most important speech feature for model development, followed by Mel-spectogram10.

## 4. Discussion

Scale score estimation results from cross-sectional and longitudinal measurements and the speech features that contributed to the model development will be discussed based on each result. Finally, we discussed the challenges and future work for this study.

### 4.1. Estimation of scale scores by cross-sectional measurement

Comparing the mean RMSE of the three scale scores among the ten algorithms, the k-NN was the best, with 13.433. The RMSE and MAE of the model with k-NN were 14.361 for the training data and 13.304 for the test data. Better test scores than the training suggest that the trained k-NN model could estimate the subscale scores on unseen subjects.

In each fold, the closer the ground truth was to the median, the lower the RMSE and MAE. On the other hand, 8 folds had RMSE and MAE above 10. Among them, fold6 had a high RMSE and MAE of 38.200 (Table 7). Subjects were unable to perform daily activities such as housework due to delusions. fold 9 had RMSE and MAE of 32.867. The subject was hospitalized in a psychiatric ward after the first measurement. In both cases, the scores of subjects who required the most medical intervention tended to deviate from the overall trend.

### 4.2. Estimation of scale scores by longitudinal measurement

Comparing the average RMSE of the three scale scores among the 10 algorithms, k-NN was the best with 13.301. The RMSE and MAE for the cross-sectional scales (“Psychosocial” 14.644, “Motivation, and Energy” 13.633, “Symptoms and Side-effects” 11.633) were above 10 for all subscales. However, the RMSE and MAE for the model with longitudinal measures were less than 10 for “Psychosocial” at 9.607, “Motivation and Energy” at 4.767, and “Symptoms and Side-effects” at 9.508.

In addition, in the model with cross-sectional measurement, 10 out of 18 folds had RMSE less than 10, while in the model with longitudinal measurement, 5 out of 6 folds had RMSE less than 10. In developing the model with the cross-sectional measurement, 1 fold was used as test data and 17 folds as training data. On the other hand, the longitudinal measurement used all data from 18 subjects as training data, which may be partly responsible for the increase in the number of training data.

### 4.3. Speech features that contributed to the estimation of scale scores

MFCC commonly contributed to the estimation of the scores of the three subscales. MFCC has many advantages, such as high discriminative power and noise immunity (39). Furthermore, MFCC can accurately characterize the vocal tract and accurately represent the phonemes produced by the vocal tract (40). The JSQLS response options are “Always,” “Often,” “Sometimes,” “Rarely,” and “Never”. Therefore, it would have been possible to use the vocalization patterns of the different choices for identification.

Finally, as issues and future perspectives of this study, the number of patients diagnosed with schizophrenia eligible to subjects was small at the collaborating institutions. Therefore, it is necessary to seek the participation of more subjects who use medical institutions and welfare services. In addition, the study found that the scores for “Symptoms and Side-effects” were the lowest among the subscales. The fact that the subjects in this study were not hospitalized patients may be a factor. Therefore, it is necessary to examine whether there is a difference in scores between hospitalized and non-hospitalized patients and to consider model building. Next, the model was developed using data from 18 subjects. Attempts were made to estimate six subjects' scale scores for the second measurement. The baseline of the scale scores differs depending on the individual conditions. Although only two measurements were used in this study, developing individual models based on continuous measurements may be helpful. In addition, it may be a more straightforward method to estimate subjective QoL by examining the possibility of estimating the scale scores using speech features of daily conversation with conversational agents (e.g., greetings).

A 3-year follow-up of schizophrenia patients in a previous study found that non-remitting patients had worse QoL and increased healthcare costs than remitting patients (41). The results of this study are considered a severe issue in psychiatric treatment in Japan, where the readmission rate is high and the length of hospital stay is extended. As one strategy, evaluating QoL using voice features enables continuous monitoring by applications and can be applied to telemedicine.

## 5. Conclusion

In this study, a model was developed to estimate the three scale scores of the Japanese Schizophrenia Quality of Life Scale (JSQLS) using speech features. The ten different machine learning algorithms were compared, with k-NN being the best. The RMSE of the training data was 14.361 and the MAE of the test data was 13.361, suggesting the generality of the model. In the estimation for scale scores on individual subjects, the RMSE and MAE were higher if the scale scores were far from the median. In this study, RMSE and MAE values were higher in subjects with psychiatric symptoms that interfered with daily life and in subjects hospitalized after the measurement. In the longitudinal measurement, a model was developed using data from 18 subjects, and scale scores were estimated for six subjects measured twice. The results showed that RF was the best, with RMSE and MAE less than ten in five of the 6 folds. The speech feature most involved in model development was MFCC, which may be the result of identifying speech patterns according to question choice. Future studies should analyze more data sets and consider model development based on longitudinal measurements of individuals.

## Data Availability

The original contributions presented in the study are included in the article/Supplementary Material. Further inquiries can be directed to the corresponding author.

## APPENDIX Hyperparameters used in model development

**Table:**

Learning Algorithms	Hyperparameters
Ridge	alpha = 1.0 tol = 0.0001
Lasso	alpha = 1.0 tol = 0.0001
Elastic Net	alpha = 1.0 tol = 0.0001 L1 ratio = 0.5
K-nearest neighbors (k-NN)	n neighbors = 5 weights = uniform leaf size = 30 metric = Minkowski power parameter for metric, *p* = 2
Decision Tree (DT)	criterion = squared error, min sample split = 2 max depth = None, min sample leaf = 1 min weight fraction leaf = 0 min impurity decrease = 0.0 ccpp alpha = 0.0
Support Vector Regression (SVR)	kernel = RBF, degree = 3, gamma = scale coef0 = 0.0, tol = 0.001, C = 1.0, epsilon = 0.1
Linear SVR (L.SVR)	epsilon = 0.0, tol = 0.0001, C = 1.0 loss = epsilon insensitive, intercept scaling = 1.0
Random Forest (RF)	n estimators = 100, criterion = squared error min sample split = 2, max depth = None min sample leaf = 1, min weight fraction leaf = 0 min impurity decrease = 0.0, ccpp alpha = 0.0
AdaBoost	estimator = Decision Tree Regressor, max depth = 3 n estimators = 50, learning rate = 1.0, loss = linear
Gradient Boosting	loss = squared error, learning rate = 0.1 n estimators = 100, subsample = 1.0 criterion = Friedman MSE, min samples split = 2 min samples leaf = 1, max depth = 3, alpha = 0.9

Tol, tolerance.

All hyperparameters are available on the Scikit learn website.
