# Corrigendum: Estimation of subjective quality of life in schizophrenic patients using speech features

**DOI:** 10.3389/fresc.2023.1219395

**Published:** 2023-06-22

**Authors:** Yuko Shibata, John Noel Victorino, Tomoya Natsuyama, Naomichi Okamoto, Reiji Yoshimura, Tomohiro Shibata

**Affiliations:** ^1^ Department of Life Science and System Engineering, Graduate School of Life Science and Systems Engineering, Kyushu Institute of Technology, Kitakyushu, Japan; ^2^ Department of Psychiatry, University of Occupational and Environmental Health, Kitakyushu, Japan

**Keywords:** quality of life, schizophrenia, speech analysis, machine learning, model development

A Corrigendum on Estimation of subjective quality of life in schizophrenic patients using speech features By Shibata Y, Victorino JN, Natsuyama T, Okamoto N, Yoshimura R and Shibata T. (2023) Estimation of subjective quality of life in schizophrenic patients using speech features. Front. Rehabil. Sci. 4:1121034. doi: 10.3389/fresc.2023.1121034

In the published article, there was an error in Table 9. The Mean values were listed as 9.940, 6.433, and 11.008 from the top, but the correct values are 9.607, 4.767, and 9.508. The standard deviation values were listed as 6.584, 7.054, and 8.575 from the top, but the correct values are 6.226, 3.990, and 5.734. The Variance values were listed from the top as 36.122, 41.470, 61.277, but the correct values are 38.757, 15.924, 32.883. In Range, “Motivation and Energy” is listed as “0.140–19.060”, but the correct range is “0.140–9.060”. In Range, “Symptoms and Side-effects” is listed as “2.770–26.050”, but the correct value is “2.770–17.050”. The correct Table 9 appears below.

**Table 9:** RMSE and MAE scores for each scale on longitudinal measurements.

Scale	Mean	Std. Dev.	Variance	Median	Range
Psychosocial	9.607	6.226	38.757	10.085	2.030–17.000
Motivation and Energy	4.767	3.990	15.924	5.020	0.140–9.060
Symptoms and Side-effects	9.508	5.734	32.883	8.465	2.770–17.050

In the published article, there was an error in Table 10. The notations were listed as “Ground truth” and “Estimated value,” but the correct terms are “First measurement” and “Second measurement”. The second measurement score (right side) for Fold 2 was listed as 62.000, 64.000, 56.000, which is corrected to 60.000, 54.000, 47.000. The RMSE and MAE for Fold 2 were listed as “20.540”, but the correct value is “13.540”. The correct table appears below.

**Table 10:** First and second measurements (ground truth), and estimation scores^a^ from longitudinal measurements.

Fold	First measurement			Second measurement			RMSE	MAE
	Psychosocial	Motivation and Energy	Symptoms and Side-effects	Psychosocial	Motivation and Energy	Symptoms and Side-effects		
1	30.000	50.000	13.000	37.000 (+7)	36.000 (+14)	22.000 (+9)	7.380	7.380
2	62.000	61.000	44.000	60.000 (−2)	54.000 (−7)	47.000 (+3)	13.540	13.540
3	47.000	57.000	34.000	40.000 (−7)	54.000 (−3)	25.000 (−9)	4.577	4.577
4	52.000	50.000	31.000	35.000 (−17)	46.000 (−4)	28.000 (−3)	6.513	6.513
5	55.000	61.000	22.000	58.000 (+3)	43.000 (−18)	16.000 (−6)	8.360	8.360
6	45.000	36.000	9.000	47.000 (+2)	43.000 (+7)	3.000 (−6)	7.393	7.393
Median	50.000	54.000	27.000	44.000	45.000	24.000		

^a^RMSE and MAE are calculated from the ground truth and estimated values of the second measurement.

**Text Correction**

In the published article, there was an error.

A correction has been made to **3. Results**, 3.4. Model comparison on test set for longitudinal measurement, Paragraph Number 1. This sentence previously stated:

“The RMSE and MAE for each subscale were 9.94 for the “Psychosocial” subscale, 6.433 for the “Motivation and Energy” subscale, and 11.008 for the “Symptoms and Side-effects” subscale (Table 9). The minimum and maximum values of RMSE and MAE for the “Symptoms and Side-effects” subscale were 2.770 and 26.050, respectively, which were larger than the other scales.”

The corrected sentence appears below:

“The RMSE and MAE for each subscale were 9.607 for the “Psychosocial” subscale, 4.767 for the “Motivation and Energy” subscale, and 9.508 for the “Symptoms and Side-effects” subscale (Table 9). The minimum and maximum values of RMSE and MAE for the “Psychosocial” subscale were 2.030 and 17.000, respectively, which were larger than the other scales.”

A correction has been made to **3. Results**, 3.4. Model comparison on test set for longitudinal measurement, Paragraph Number 2. This sentence previously stated:

“Fold 3 had the lowest RMSE and MAE at 4.557 and fold 2 had the highest at 20.540.”

The corrected sentence appears below:

“Fold 3 had the lowest RMSE and MAE at 4.557 and fold 2 had the highest at 13.540.”

A correction has been made to **4. Discussion**, 4.2. Estimation of scale scores by longitudinal measurement, Paragraph Number 1. This sentence previously stated:

“However, the RMSE and MAE for the model with longitudinal measures were less than 10 for “Psychosocial” at 9.940 and “Motivation and Energy” at 6.4334.”

The corrected sentence appears below:

“However, the RMSE and MAE for the model with longitudinal measures were less than 10 for “Psychosocial” at 9.607, “Motivation and Energy” at 4.767, and "Symptoms and Side-effects” at 9.508.”

The authors apologize for these errors and state that this does not change the scientific conclusions of the article in any way. The original article has been updated.

